# Sleep Ameliorating Effects of Acupuncture in a Psychiatric Population

**DOI:** 10.1155/2013/969032

**Published:** 2013-05-27

**Authors:** Peggy Bosch, Gilles van Luijtelaar, Maurits van den Noort, Sabina Lim, Jos Egger, Anton Coenen

**Affiliations:** ^1^Donders Centre for Cognition, Radboud University Nijmegen, Postbus 9104, Montessorilaan 3, 6500 HR Nijmegen, The Netherlands; ^2^LVR-Klinik Bedburg-Hau, Bahnstrasse 6, 47551 Bedburg-Hau, Germany; ^3^Division of Acupuncture & Meridian, WHO Collaborating Center for Traditional Medicine, East-West Medical Research Institute and School of Korean Medicine, Kyung Hee University, Number 1 Hoegi-Dong, Dongdaemoon-ku, Seoul 130-701, Republic of Korea; ^4^TALK, Free University of Brussels, Pleinlaan 2, 1050 Brussels, Belgium; ^5^Behavioural Science Institute, Radboud University Nijmegen, Montessorilaan 3, 6525 HR Nijmegen, The Netherlands; ^6^Centre of Excellence for Neuropsychiatry, Vincent van Gogh Institute for Psychiatry, Stationsweg 46, 5803 AC Venray, The Netherlands

## Abstract

The interest of psychiatric patients for complementary medicine, such as acupuncture, is stable, but effect studies in psychiatry remain scarce. In this pilot study, the effects of 3 months of acupuncture treatment on sleep were evaluated and compared between a group of patients with schizophrenia (*n* = 16) and a group with depression (*n* = 16). Healthy controls were included in order to establish reference values (*n* = 8). Patients with schizophrenia and depression were randomly assigned to either a waiting list or a treatment condition. The Pittsburgh Sleep Quality Inventory was completed before and after the acupuncture treatment (individualized and according to traditional Chinese medicine principles) or the waiting list condition. Both acupuncture groups showed significant lower scores on the sleep inventory, which was not the case for the waiting list condition. Moreover, it was found that the effectiveness of the acupuncture treatment was higher in the patients with schizophrenia than in the patients with depression. Acupuncture seems able to improve sleep in this convenient sample of patients with long-lasting psychiatric problems and may be a suitable and cost-effective add-on treatment for this group, particularly if conducted group-wise.

## 1. Introduction

Interest in complementary and alternative medicine (CAM), such as acupuncture, has increased in popularity in Western societies in the last part of the twentieth century and there has been a continued interest ever since [1]. The use of CAM includes its application in psychiatric patients [2]. Two large groups that need long-term treatment are patients with depression and patients with schizophrenia. Besides the typical depressive or positive and negative symptoms, their disorders are characterized by marked disturbances of sleep [3–8]. Patients with schizophrenia show increased sleep latency, decreased total sleep time, and decreased sleep efficiency [3–5]. A bidirectional relationship between insomnia and depressive symptoms in patients with depression is described [6–8]. Sleep problems such as increased sleep latency, awakenings in the night or early in the morning with an incapability to go back to sleep, and decreased sleep efficiency are typical symptoms of depression, whereas hypersomnia and dream disturbances are also often reported. The sleep disorders appear to maintain or even deteriorate the mood disorder [9].

In Western psychiatry, a growing consumption of antipsychotic [10] and antidepressant drugs [11] can be seen. Antipsychotic application is seen as the cornerstone in therapy for patients with schizophrenia [12], whereas due to their positive effects, antidepressants have found their place in the clinical guidelines for the treatment of, for instance, patients with depression [13]. Despite these clinical successes, a problem with pharmaceutical therapies [14], like any other therapy, is that they are subject to patient nonadherence and declining patient compliance [15, 16].

Previous research has shown that adherence to treatment correlates negatively with sleep disturbance and depression [17]. It seems that a lack of contact with this patient group makes it difficult to engage in a therapeutic relationship or to prescribe and monitor medication effectively. The medicines used to treat these conditions often cause drowsiness [18]. Patients are therefore often advised to take them at night which causes problems with sleep (e.g., excessive dreaming and increase in total sleep time in patients with schizophrenia [4]), even though taking them at night minimizes daytime drowsiness. Moreover, tiredness, drowsiness, and poor sleep interfere with the patient's ability to engage with therapeutic services because they are too tired, unmotivated and they do not see the point or do not want to take medicines that cause such adverse effects. Presumably, these are not the only factors that are of importance, but they seem highly relevant in patient groups that suffer from depression, as well as those that suffer from schizophrenia, since both diseases are prone to sleep disorders.

Acupuncture is part of Traditional Chinese Medicine (TCM), which in itself is a form of CAM that is based on thousands of years of practice [21]. One of the features of the TCM approach is the individualization of treatment, which relies on a symptom-based diagnostic process [22]. TCM diagnoses are based on clinical symptoms and signs that are completely discerned by the oriental medical practitioner [22].

Recent years have seen an increase in trials on particularly depression and acupuncture [23–25]. Various reviews, however, have concluded that evidence for the effects of acupuncture on depression still remains preliminary [26–28]. In particular methodological problems, such as different techniques (electro-, manual, or laser acupuncture), various control groups and study designs limit the generalization of the results [23, 29]. Although efforts have been made to standardize and optimize research and the way it is reported, further research is warranted [26, 30].

Even less research with acupuncture has been conducted on schizophrenia. Some literature research [31, 32] indicates that more research is necessary to draw firm conclusions on acupuncture's effectiveness in the treatment of schizophrenia. Moreover, even though some research was reported [33, 34], hinting in the direction of effectiveness and thereby providing the basis for future research, the existing research remains preliminary.

Acupuncture may be beneficial in the treatment of sleep disorders [35]. It can be used alone or combined with other interventions, since no interactions were found to date and adverse events related to treatment seem sufficiently controllable by providing thorough training [36]. Although some research has been conducted on acupuncture and sleep disorders [37–40], results are still tentative, particularly in patients with psychiatric disorders. Results call for research in a group, in which symptoms are prominent, since acupuncture's effectiveness is thought to rely on its homeostatic actions, striving to return the body to its normal physiological state. Therefore, it is thought that acupuncture has more effects on patients that experience serious problems than on healthy participants or patients with only mild symptoms [41].

This pilot study evaluates and compares the effects of acupuncture on the subjective quality of sleep in long-term patients with schizophrenia and patients with depression. It is a pragmatic trial and a first start to conduct research in an integrative treatment setting in which psychiatric treatment and TCM are combined.

## 2. Materials and Methods

### 2.1. Participants

In total the convenient sample consisted of 40 participants (13 men, 27 women). Sixteen of them (10 women, 6 men, mean age was 44.25 years, SD = 2.44) were diagnosed with schizophrenia, 16 of them (12 women, 4 men, mean age was 50.94 years, SD = 1.33) were diagnosed with depression. The healthy control group consisted of 5 women and 3 men (mean age was 36.75 years, SD = 12.43). Mean length of illness was 13.56 years (SD = 1.59) for the group with schizophrenia and 5.94 years (SD = 1.05) for the group with depression. The randomization function in Excel was used to randomly divide the patients into a treatment and a waiting list condition. For an overview of the descriptive statistics see Table 1 and for an overview of the medication used see Table 2.

Recruiting limitations resulted in a higher mean age in the depression waiting list condition than in the healthy control group (*P* < 0.05). There was a poster in the entrance section and in the waiting room that gave information on the study. Moreover, potential participants (diagnosed with schizophrenia F20.0 (paranoid schizophrenia), F20.5 (schizophrenic residuum), or depression F33.2 according to the ICD-10) [42] were identified and approached by their therapist at the LVR-Klinik Bedburg-Hau. Patients that agreed to participate did so voluntarily and signed an informed consent form; moreover, their therapist signed for their mental ability to understand the form. The Becks Depression Inventory-II [43] and Positive And Negative Symptom Scale (PANSS) [44] were used as inclusion criteria. The study was carried out in accordance with the Declaration of Helsinki [45] and was approved by the ethical committee of the Ärztekammer Nordrhein. Participants continued with their normal psychiatric treatment, including medication, alongside acupuncture. After the project, the medical files were checked for possible changes in medication during the time of the project. Moreover, possible use of sleep medication was mentioned by the patients on the Pittsburgh Sleep Quality Inventory (PSQI). Five of the patients with schizophrenia (all in the acupuncture condition) used sleep medication beforehand. Six of the patients with depression (2 in the acupuncture and 4 in the waiting list condition) and none of the healthy control group used sleep medication. Of the five patients in the schizophrenia and acupuncture group, one of them used Prothipendyl (80 mg daily), one used Prothipendyl (40 mg daily), one used Sifrol (0.36 mg daily) one used Amitriptyline (50 mg daily), and one used Melperone (75 mg daily). In the depression and acupuncture group one patient used Chlorprothixene (30 mg daily) and one patient Pipamperone (40 mg daily). Finally in the depression and waiting list condition group one patient used Pipamperone (40 mg daily) and three patients Zopiclone (7.5 mg daily/when needed). Probably due to the natural course of the diseases and recruitment limitations, the group of participants diagnosed with schizophrenia had been in treatment significantly longer than those with depression. There were no gender differences within and between the groups. Exclusion criteria for the patients were substance abuse and/or epilepsy and other neurological conditions. For the control group, the exclusion criteria were the presence of neurological or psychiatric disorders.

### 2.2. Instruments

#### 2.2.1. Pittsburgh Sleep Quality Inventory

The German version of the PSQI [46] was used in order to gain information on the subjective quality and quantity of sleep in the participants. This retrospective list contains questions about sleep during the last four weeks, information on the number of sleep disturbances, estimation on sleep quality, sleep duration, sleep latency and sleep times, use of medication, and sleepiness during the day. The questionnaire consists of 18 items, divided into 7 components that can be scored from 0 to 3. The PSQI Total Score results in the sum of the component scores and can be any score from zero to 21. A high score means sleep quality is bad. Five was originally seen as the cutoff score. Participants that score below 5 have a good sleep quality. However, there is a tendency to use 6 as a cutoff score [47] to be more selective. The internal consistency for the American and Japanese versions was found to be good. Cronbach's alpha for the total score was .77 [48]. The validity of the PSQI in patients with primary insomnia was good, since a high correlation between PSQI scores and a sleep diary was obtained by Backhaus and colleagues [49]. Moreover, they found a significant correlation between the PSQI and polysomnographic measurements. These results were confirmed in research on the Chinese version of the PSQI [50]. In all, the PSQI has a high sensitivity and specificity for patients with insomnia [49] and also for patients with depression and schizophrenia [48].

### 2.3. Experiment

#### 2.3.1. Needles

The needles (AcuPro C, Wujiang City Cloud & Dragon Medical Device Co., Ltd., China) that were used were 0.25 × 25 mm or 0.20 × 15 mm stainless steel (depending on the place of needling) single-use needles. The needles were placed according to TCM principles.

#### 2.3.2. Intervention

The participants in the acupuncture groups were given acupuncture treatment once a week, twelve weeks in a row. Individualized acupuncture according to TCM principles was applied after careful individual diagnosis by a licensed oriental medical practitioner with more than 5 years of clinical experience [30]. Acupuncture treatment took place in a light room with (very) soft background music (Enya) playing. According to the demand of the ethics committee to decrease anxiety [51] as much as possible in patients with schizophrenia and to make them feel comfortable, music along with acupuncture was used. The music was kept constant over all participants and all sessions. There were 12 “relax” chairs in which it was possible to adjust the back and put the feet up, resulting in a near-lying position. It was, however, also possible to remain upright; this was left to the patients to decide for themselves. Due to the fact that acupuncture was applied with patients in a sitting or near-lying position, access to acupuncture points on the back was limited. Patients came into the room in intervals, in order to reduce waiting time. Treatment (after needles were inserted) lasted for one hour. After this hour, needles were removed. This group treatment setting made sure practitioners were directly at hand in case anxiety would arise and it was one of the important points that were made in the dialogue with the ethics committee. In case individuals had personal questions or sensitive matters that needed to be discussed prior to treatment, there was an empty room next to the treatment room where confidentiality could be assured. As there were two acupuncturists present, the other patients would not be left alone in the mean time.

All participants continued with regular treatment including appointments with their psychiatrists; this was not influenced by the project since acupuncture was used as an add-on treatment.

#### 2.3.3. Procedure

All participants were tested in an experimental testing room in the clinic, by apprentices who were blind to group or time of testing. The healthy control group was tested at T1 (pretest) only. The participants with schizophrenia and depression were tested at T1 and T2 (posttest). After the tests at T1, participants were randomly divided into a treatment and a waiting list condition. The duration of the whole experiment was 13 weeks, which included 12 weekly acupuncture sessions and pre- and post-testing. At the end of the experiment, all participants received a debriefing and were individually informed about their own test results. Patients on the waiting list were given the opportunity to attend acupuncture treatment after T2 if they wanted to. The current study stopped at T2, although acupuncture treatment was given after T2 in order to provide equal treatment opportunities. The patients, however, were not tested afterwards and therefore these data were not included in the study. Moreover, any acupuncture that was provided after the study was part of their normal treatment, not of any study.

### 2.4. Statistics

Differences between the five groups on the subtests of the PSQI before the start of the treatment were analyzed with a one-way analysis of variance (one-way ANOVA) with groups as between subjects factor, followed by posthoc (Bonferroni) tests. Repeated measures analyses of variance were used to analyze possible differences on the PSQI Total Score and on the subtests of the PSQI pre- and posttreatment (in the four experimental groups), followed by posthoc (Bonferroni) *t*-tests if appropriate, that is, *t*(7) in our pilot-study. A value of *P* < 0.05 was considered to be statistically significant.

## 3. Results

### 3.1. Acupuncture Points That Were Used

For more details see Table 3.

### 3.2. Pretest Results

Descriptive characteristics of the five different groups are shown in Table 4, as well as the outcomes of the posthoc tests following the one-way ANOVA. On some of the subtests differences were found between the healthy control and the psychiatric groups.

### 3.3. Evaluation of Sleep Quality between the Groups

All patients randomized and treated over 12 weeks in the depression and schizophrenia groups were analyzed (each *n* = 8). As can be seen in Table 5, post hoc *t*-tests for each subtest and group separately resulted in the following significant differences: the depression acupuncture group showed a significant reduction for PSQI Total Score* t*(7) = 4.333, *P* = 0.003. For the depression waiting list condition, no significant differences were found between the pre- and posttest measurements. A significant reduction for the schizophrenia acupuncture group was found for PSQI Total Score *t*(7) = 2.393, *P* = 0.048, for PSQI Latency *t*(7) = 2.553, *P* = 0.038, for PSQI Disorders *t*(7) = 2.646, *P* = 0.033, and for PSQI Medicine *t*(7) = 2.646, *P* = 0.033. No differences were found for the schizophrenia waiting list condition.

### 3.4. Side Effects

Two patients reported bruising as a side effect after one of the acupuncture treatment sessions. Moreover, one patient reported having been extremely tired after the first session. Otherwise, no side effects were reported.

## 4. Discussion

In this pilot study, the effects of three months of acupuncture treatment on subjective sleep quality were investigated in a group of patients with schizophrenia and a group of patients with depression that were diagnosed by their therapists according to the ICD-10 [42]. All patients were chronically ill. Significant improvements were found on the PSQI Total Score for both treatment groups, indicating that patients slept better after 12 acupuncture treatments. The waiting list condition groups showed no significant improvements. As was suggested by Hametner and colleagues [47], a cutoff score of 6 can be used in order to clinically divide patients with sleep problems from patients with good sleep. The patient group with schizophrenia falls below this clinically relevant score after treatment. The patient group with depression has improved and although the differences might not seem large, they seem borderline clinically relevant.

Three subscales (PSQI Latency, PSQI Medication, and PSQI Disorders) showed significant improvements in the schizophrenia group, but not in the depression group. This indicates that the patients with schizophrenia took more benefit from acupuncture than the patients with depression. Of note, these patients fell asleep faster and even approached normal levels on the subtest (PSQI Latency), meaning that patients with schizophrenia lay awake less long before falling asleep after acupuncture treatment and that they reached levels that are commonly found in healthy controls. They also used less medication in order to sleep and reached normative levels also on the subtest for sleep disorders. Five of the patients with schizophrenia (from the acupuncture condition) used sleep medication of different kinds beforehand, whereas four of them answered that they had stopped using this medication during the time of the acupuncture treatment. Moreover, one of the patients in the waiting list condition of this group, who had not used sleep medication beforehand, started using sleep medication. On the other hand, six of the patients with depression (two in the acupuncture group and four in the waiting list condition) used sleep medication beforehand of which 4 (in the waiting list condition) stopped using this medication and one of the other patients in the waiting list group started to use sleep medication. There were no differences between or within the depression groups on medication use as reported by the patient.

The intervention phase lasted three months (12 treatments) only. Future studies might consider whether the novelty factor of this intervention or the short-term availability implies that patients are more likely to attend. It is not known whether patients would be so keen to attend acupuncture were it available as part of their normal treatment package. There were no withdrawals from the acupuncture or waiting list groups in this study. In this clinic, as part of the normal treatment package, patients can choose to visit treatment groups like, for instance; a psychosis education group, sleep training, depression group, social competency training group or a memory training. All of these groups last 10 to 12 times and have a dropout rate between 30 to 40%. These differences between the regular groups and this study might be caused by the small amount of appointments in the waiting list condition as well as a positive experience in the acupuncture groups. This impression is supported by the absent dropout and the comments made by participants (that reported, for instance: feeling less tired, more relaxed, and better able to sleep), that they were satisfied with the treatment and keen to have it. On the other hand, it is important to note that the participants were largely self-selecting (as they are in every group they attend in this clinic) and therefore more likely to come to the treatments anyway. However, in order to draw more firm conclusions, it would have been better to implement a measure of treatment satisfaction in the study.

Some participants reduced their medication, in consultation with their psychiatrist, as a result of the acupuncture treatment. These participants saw this as a benefit of the acupuncture. Medication reduction is usually seen as positive by patients. It is felt to be a sense of improvement or achievement. It may be that the promise of a reduction in medication through acupuncture may be a motivational factor for attendance at acupuncture treatments. On the other hand, it is important to note that there are possible pitfalls in reducing medication as well. It has been described that patients with schizophrenia who improve through the use of acupuncture and as a result reduce or even stop taking medication may become more vulnerable to breakdown [52]. Further research is needed to confirm these subjective comments that were reported by the patients in this study and to investigate the possibility that acupuncture may be misused as an excuse for nonadherence with conventional medication.

*Limitations.* Since the study involves acupuncture, it is obvious that the problem of the absence of a suitable control group or placebo needs to be mentioned [30]. In this study, it was chosen to investigate the “normal” or “real-world” manual individualized acupuncture treatment that any patient would receive if they should go to an oriental medical practitioner. The use of a standardized protocol for acupuncture is, besides the National Acupuncture Detoxification Association (NADA) protocol that is used for addiction and trauma [53], unheard of in clinical TCM practice. The use of such a standardization would therefore not shed any light on the possible effect that an acupuncture add-on treatment (that patients seek outside our psychiatric clinics) would have on patients and would not be generalisable to routine clinical practice [54]. In this study, a pragmatic randomized controlled trial (RCT) was used; this approach attempts to answer a “real-world” question whether acupuncture as add-on treatment improves sleep more than without this treatment. Our overall goal was to deliver better treatment to patients and this implies that we have to evaluate what can be done in daily practice. MacPherson [55] paraphrased this issue by stating that “the question in acupuncture research should be rather whether acupuncture is of better value than what is currently on offer instead of asking whether acupuncture is better than placebo?”

Due to the ethical problems related to discontinuing treatment with antipsychotic and antidepressant drugs, patients continued their medication during the study. We have listed the doses in Chlorpromazine equivalents and information on medication that was used in Table 2. Due to the fact that psychiatric patients use a wide variety of medication, it was not possible, within the convenient sample in our monocenter pilot study, to include only those that use the same medication and medication doses.

One more limitation of the study is the fact that a second baseline might have been used; it is recommended for future research.

Since the ethics committee required group treatments due to the fact that a practitioner needed to be present at all times, a limitation was that some participants talked to each other before, during, or after treatment sessions. It was not possible to control for the content of these conversations.

Finally, the number of patients in the present study is relatively small. Therefore, in further research it is necessary to increase the sample size, though, despite the small numbers, significant improvements in sleep quality were found.

There is anxiety about giving acupuncture to people with schizophrenia in Europe, since it is not normally practiced and people in psychiatric hospitals are not normally left alone with needles or other dangerous objects. Moreover, anxiety exists about the needles becoming part of hallucinations or psychotic thoughts. For instance, patients might think that they are being radiographic controlled through the needles. The present study further proves that people with schizophrenia can be safely treated with acupuncture and that the use of needles did not evoke negative emotional reactions.

It is important to realize that in this pilot study, positive results were obtained in a group of patients with schizophrenia that have been ill for more than 10 years. Length of illness was analyzed more specifically and it was found that, although there was a difference between the schizophrenia and depression experimental and waiting list groups when it comes to this factor, it did not account for the more significant results in the group with schizophrenia.

It is obvious that the positive outcomes of this pilot study warrant further and larger-scale research, but the tentative conclusion is that the present study shows that acupuncture seems to influence sleep in a positive way in sleep-disturbed patients and seems a suitable add-on treatment in psychiatry, even in patients with long-term depression or schizophrenia.

## Figures and Tables

**Table 1:** Overview of the descriptive statistics of the convenient sample.

		Schizophrenia (SD)			Depression (SD)		Healthy control (SD)
	Total	Waiting list	Acupuncture	Total	Waiting list	Acupuncture	Total
Men	6	3	3	4	2	2	3
Women	10	5	5	12	6	6	5
Length of illness	13.56 (1.59)	12.63 (5.90)^a^	14.50 (7.07)^a^	5.94 (1.05)	4.38 (3.54)	7.50 (4.41)	0
Age	44.25 (2.44)	42.25 (10.99)	46.25 (8.57)	50.94 (1.33)	52.88 (5.59)^b^	49.00 (4.54)	36.75 (12.43)

According to the one-way ANOVA (groups as between subjects factor) at baseline: ^a^Mean is significantly different (*P* < 0.05) from the depression waiting list group. ^b^Mean is significantly different (*P* < 0.05) from the healthy control group.

**Table 2:** Overview of the medication used by the different groups at the start of the study.

Group	CPZ	Atypical	Typical	SSRI	Tricyclic antidepressives	SNRI and SSNRI	Others
Depression and acupuncture group	Chlorprothixene in 1 patient Promethazine in 1 patient Pipamperone in 1 patient 0.33 in 1 patient	In 2 patients	In 2 patients	In 4 patients	In 3 patients	In 3 patients	In 2 patients

Depression and waitlist group	Pipamperone in 1 patient 0.33 in 1 patient	In 1 patient	In 1 patient	In 4 patients	In 2 patients	In 3 patients	In 2 patients

Schizophrenia and acupuncture group	Amisulpride + 1 in 1 patient Zotepine + 1 in 1 patient Prothipendyl + 2 in 1 patient Fluphenazine + 3 in 1 patient 6 in 1 patient 4 in 1 patient 3.5 in 1 patient 1.83 in 1 patient	In 8 patients	In 3 patients	In 0 patients	In 1 patient	In 1 patients	In 4 patients

Schizophrenia and waitlist group	Fluphenazine + 3 in 1 patient 6 in 1 patient Pipamperone + 3.17 in 1 patient 1.83 in 1 patient 1 in 1 patient Zotepine and Chlorprothixene and Melperone + 4 in 1 patient 10 in 1 patient 3.5 in 1 patient	In 8 patients	In 3 patients	In 0 patients	In 2 patients	In 0 patients	In 4 patients

CPZ (Chlorpromazine Equivalents) were calculated using published equivalencies for oral conventional [19] and atypical [20] antipsychotics.

SSRI: selective serotonin reuptake Inhibitor, SNRI: serotonin norepinephrine reuptake inhibitor, SSNRI: selective serotonin norepinephrine reuptake inhibitor.

**Table 3:** Acupuncture points that were used.

Points/patients	D1	D2	D3	D4	D5	D6	D7	D8	S1	S2	S3	S4	S5	S6	S7	S8
EX-HN-1	12	12	12	12	12	12	11	12	12	11	12	12	11	12	12	12
DU-24									2	5	5	2				8
DU-14										1						
DU-17									1							
DU-18									1							1
DU-19									1							
EX-HN-3	5		1			2										
EX-HN-5	5		1			2									1	
LI-20						1										
ST-8	10	3	3	9	8		7	2		7	5		10	1	2	1
ST-7									1							
ST-6					2											
TB-21							1						1			
KI-23						2										
KI-24					1	2			1							
KI-25					1				1							
KI-26									1							
GB-6	1															
GB-7									1							
GB-8									1	1						
GB-13									2	1	2		1			9
GB-20	2				1											
SI-3	5		11							2	2		1			
SI-4												1				
SI-5															2	
HT-2					1											
HT-3	1	4	3		3	1	4	2								
LI-4	12	12	12	12	12	12	12	12	7	3	5	9	10	8	12	4
PC-6	3	2		1	4	2			1	1		2	1	1	1	
PC-7		1			4			2	5	4	2	1	2		1	7
HT-7	12	12	11	12	12	12	12	12	9	1	7	3	3	8	3	
HT-8					1											
LU-5												1			1	
LU-6													8			
LU-7	1	11	8		5	3	3	12	8		1		3	5	1	
TB-5	5		10	3			2						2			
TB-6												1				
LI-7	1	2	1			2	10		2	1		1	1			
LI-11	6	6	3	12	10	1	12	6	5	10	8	8	8	11	12	11
CV-12																2
CV-14									1							
CV-15								1								
CV-16					1								1			
CV-17	7	2	3	7	10	1	7	9		12	3	6	9	9	8	11
CV-18					2									3	1	
ST-21										3						4
ST-25	4								2	1		3	5		1	3
CV-5											1					
CV-4	3	8	9		2			9								
KI-10			1	2												
SP-10	7	6	9			2	3	1				1				
BL-39													1			
BL-40													1	3		
SP-9	12	12	12	12	11	12	12	12	7	6	10	9	12	12	12	3
GB-34	8	7	5	7	8	6	5	6	1			1				4
ST-36	12	12	12	8	12	12	2	12	7	1	3	8	9	8	9	2
ST-40	1	1		12			11		1	10	9	3	2	4	2	9
SP-6	12	12	12	12	12	12	12	11	11	4	12	8	12	11	12	10
KI-3	11	12	12	12	12	12	12	12	1	2	4	7	11		12	
KI-5														2		
KI-6	9	8	9			5	8	12	2							2
LR-3	10	12	11	12	10	4	7	10	10	9	6	8	10	9	5	7
LR-1	2			2	4											
SP-4	4	4	1	11	4	7	9	7	1			2				
BL-60			2					2					1	3		
BL-62													2			
ST-44									2	7			1	3		7
ST-45				6			2				1			1		
GB-41												1				1
GB-44	3			9	6		2		1	5				4		2
GB-45														1		
BL-67	1	1			6	5		3	2		1	1	11	1		
Eye of the knee						8		3		5				6		
BAXIE						3										

D: patient with depression, S: patient with schizophrenia.

**Table 4:** Corrected means and SDs of the PSQI subtest scores at baseline (T1) for all groups.

PSQI subtest	Schizophrenia	Schizophrenia	Depression	Depression	Healthy (SD)
	waiting list (SD)	acupuncture (SD)	waiting list (SD)	acupuncture (SD)	
Total score	5.75 (1.91)	8.50^b^ (4.21)	9.63^b^ (4.57)	8.50^b^ (3.02)	3.50 (2.07)
Subjective sleep quality	1.00 (0.76)	1.00 (0.76)	1.63 (0.52)	1.38 (0.52)	0.75 (0.46)
Latency	0.87 (1.36)	1.88 (1.13)	1.50 (1.07)	1.50 (0.93)	0.50 (0.54)
Duration	0.25 (0.46)	0.38 (1.06)	1.00 (1.41)	0.50 (0.93)	0.63 (0.74)
Efficiency	1.38 (1.51)	1.00 (1.07)	1.13 (1.55)	1.00 (1.31)	0.25 (0.71)
Disorders	0.88 (0.35)	1.38 (0.52)	1.63 (0.74)	1.50 (0.54)	0.88 (0.35)
Medication	0.00^a^ (0.00)	1.88^b^ (1.55)	1.25 (1.49)	0.75 (1.39)	0.00 (0.00)
Daytime sleepiness	1.38 (0.52)	1.00^b^ (0.76)	1.50 (0.93)	1.88 (0.84)	0.50 (0.54)

According to the one-way ANOVA (groups as between subjects factor) and post hoc tests at baseline: ^a^Mean is significantly different (*P* < 0.05) from the schizophrenia acupuncture group. ^b^Mean is significantly different (*P* < 0.05) from the healthy control group.

**Table 5:** Corrected pretest (T1) means of the PSQI for all five groups and posttest (T2) means of the PSQI for the four groups with patients.

PSQI subtest	Schizophrenia waiting list			Schizophrenia acupuncture			Depression waiting list			Depression acupuncture			Healthy control
	T1	T2	*P*	T1	T2	*P*	T1	T2	*P*	T1	T2	*P*	T1
Total score	5.75	4.88	0.576	8.50	5.50	0.048*	9.63	9.00	0.493	8.50	6.88	0.003**	3.50
Subjective quality	1.00	0.75	0.170	1.00	0.50	0.170	1.63	1.50	0.685	1.38	1.00	0.080	0.75
Latency	0.87	0.75	0.732	1.88	0.75	0.038*	1.50	1.63	0.685	1.50	1.12	0.197	0.50
Duration	0.25	0.13	0.598	0.38	0.63	0.351	1.00	0.75	0.451	0.50	0.63	0.351	0.63
Efficiency	1.38	0.63	0.365	1.00	1.50	0.407	1.13	1.38	0.563	1.00	0.75	0.170	0.25
Disorders	0.88	0.88	1.00	1.38	0.88	0.033*	1.63	1.63	1.00	1.50	1.13	0.080	0.88
Medication	0.00	0.38	0.351	1.88	0.38	0.033*	1.25	0.13	0.094	0.75	0.75	1.00	0.00
Daytime sleepiness	1.38	1.38	1.00	1.00	0.88	0.685	1.50	2.00	0.104	1.88	1.50	0.080	0.50

Difference T1-T2 within the groups: *P < 0.05, **P < 0.005.

## References

[1] Harris PE, Cooper KL, Relton C, Thomas KJ (2012). Prevalence of Complementary and Alternative Medicine (CAM) use by the general population: a systematic review and update. *International Journal of Clinical Practice*.

[2] Elkins G, Rajab MH, Marcus J (2005). Complementary and alternative medicine use by psychiatric inpatients. *Psychological Reports*.

[3] Chouinard S, Poulin J, Stip E, Godbout R (2004). Sleep in untreated patients with schizophrenia: a meta-analysis. *Schizophrenia Bulletin*.

[4] Monti JM, Monti D (2004). Sleep in schizophrenia patients and the effects of antipsychotic drugs. *Sleep Medicine Reviews*.

[5] Monti JM, Monti D (2005). Sleep disturbance in schizophrenia. *International Review of Psychiatry*.

[6] Buysse DJ, Angst J, Gamma A, Ajdacic V, Eich D, Rössler W (2008). Prevalence, course, and comorbidity of insomnia and depression in young adults. *Sleep*.

[7] Cukrowicz KC, Otamendi A, Pinto JY, Bernert RA, Krakow B, Joiner TE (2006). The impact of insomnia and sleep disturbances on depression and suicidality. *Dreaming*.

[8] Taylor DJ, Lichstein KL, Durrence HH, Reidel BW, Bush AJ (2005). Epidemiology of insomnia, depression, and anxiety. *Sleep*.

[9] Stein DJ, Kupfer DJ, Schatzberg AF (2006). *The American Psychiatric Publishing Textbook of Mood Disorders*.

[10] Lertxundi U, Echaburu DS, Palacios HR (2012). The use of antipsychotics in a medium-long stay psychiatric hospital from 1998 to 2010. *International Journal of Psychiatry in Clinical Practice*.

[11] Pratt LA, Brody DJ, Gu Q (2011). Antidepressant use in persons aged 12 and over: United States, 2005–2008. *NCHS Data Brief*.

[12] Tandon R, Nasrallah HA, Keshavan MS (2010). Schizophrenia, “Just the Facts” 5. Treatment and prevention Past, present, and future. *Schizophrenia Research*.

[13] National Institute for Health and Clinical Excellence (NICE) (2010). Depression: the NICE guideline on the treatment and management of depression in adults. *National Collaborating Centre For Mental Health For the National Institute For Health & Clinical Excellence*.

[14] Ascher-Svanum H, Peng X, Faries D, Montgomery W, Haddad PM (2009). Treatment patterns and clinical characteristics prior to initiating depot typical antipsychotics for nonadherent schizophrenia patients. *BMC Psychiatry*.

[15] Bodén R, Brandt L, Kieler H, Andersen M, Reutfors J (2011). Early non-adherence to medication and other risk-factors for rehospitalization in schizophrenia and schizoaffective disorder. *Schizophrenia Research*.

[16] Goff DC, Hill M, Freudenreich O (2010). Strategies for improving treatment adherence in schizophrenia and schizoaffective disorder. *Journal of Clinical Psychiatry*.

[17] Phillips KD, Moneyham L, Murdaugh C (2005). Sleep disturbance and depression as barriers to adherence. *Clinical Nursing Research*.

[18] Seeman MV (2012). Antipsychotic-induced somnolence in mothers with schizophrenia. *Psychiatric Quarterly*.

[19] American Psychiatric Association (1997). Practice guideline for the treatment of patients with schizophrenia. *American Journal of Psychiatry*.

[20] Woods SW (2003). Chlorpromazine equivalent doses for the newer atypical antipsychotics. *Journal of Clinical Psychiatry*.

[21] Kavoussi B (2007). Chinese medicine: a cognitive and epistemological review. *Evidence-Based Complementary and Alternative Medicine*.

[22] Zhang GG, Lee W, Bausell B, Lao L, Handwerger B, Berman B (2005). Variability in the Traditional Chinese Medicine (TCM) diagnoses and herbal prescriptions provided by three TCM practitioners for 40 patients with rheumatoid arthritis. *Journal of Alternative and Complementary Medicine*.

[23] Yeung AS, Ameral VE, Chuzi SE, Fava M, Mischoulon D (2011). A pilot study of acupuncture augmentation therapy in antidepressant partial and non-responders with major depressive disorder. *Journal of Affective Disorders*.

[24] Lyons Z, van der Watt G, Shen Z, Janca A (2012). Acupuncture and Chinese herbs as treatments for depression: an Australian pilot study. *Complementary Therapies in Clinical Practice*.

[25] Zhang WJ, Yang XB, Zhong BL (2009). Combination of acupuncture and fluoxetine for depression: a randomized, double-blind, sham-controlled trial. *Journal of Alternative and Complementary Medicine*.

[26] Leo RJ, Ligot JSA (2007). A systematic review of randomized controlled trials of acupuncture in the treatment of depression. *Journal of Affective Disorders*.

[27] Schroer S, Adamson J (2011). Acupuncture for depression: a critique of the evidence base. *CNS Neuroscience Therapy*.

[28] Wang H, Qi H, Wang BS (2008). Is acupuncture beneficial in depression: a meta-analysis of 8 randomized controlled trials?. *Journal of Affective Disorders*.

[29] Bosch MPC, Ausfeld B, van den Noort MWML, Bosch MPC, van den Noort MWML (2008). Acupuncture modalities, methodology, and key problems for western scientific research. *Schizophrenia, Sleep, & Acupuncture*.

[30] MacPherson H, White A, Cummings M, Jobst KA, Rose K, Niemtzow RC (2010). Revised Standards for reporting interventions in controlled trials of acupuncture: extending the CONSORT Statement. *PLoS Medicine*.

[31] Bosch MPC, van den Noort MWML (2008). *Schizophrenia, Sleep, and Acupuncture*.

[32] Rathbone J, Xia J (2005). Acupuncture for schizophrenia. *Cochrane Database of Systematic Reviews*.

[33] Lee MS, Shin BC, Ronan P, Ernst E (2009). Acupuncture for schizophrenia: a systematic review and meta-analysis. *International Journal of Clinical Practice*.

[34] Ronan P, Harbinson D, MacInnes D, Lewis W, Robinson N (2010). Acupuncture and schizophrenia-effect and acceptability: preliminary results of the first UK study. *European Journal of Oriental Medicine*.

[35] Peterson JR (2002). Acupuncture and the treatment of insomnia. *Medical Acupuncture*.

[36] Vincent C (2001). The safety of acupuncture: acupuncture is safe in the hands of competent practitioners. *British Medical Journal*.

[37] Guerreiro da Silva JB, Nakamura MU, Cordeiro JA, Kulay L (2005). Acupuncture for insomnia in pregnancy-a prospective, quasi-randomised, controlled study. *Acupuncture in Medicine*.

[38] Freire AO, Sugai GCM, Chrispin FS (2007). Treatment of moderate obstructive sleep apnea syndrome with acupuncture: a randomised, placebo-controlled pilot trial. *Sleep Medicine*.

[39] Montakab H (1999). Acupuncture and insomnia. *Forschende Komplementarmedizin*.

[40] Spence DW, Kayumov L, Chen A (2004). Acupuncture increases nocturnal melatonin secretion and reduces insomnia and anxiety: a preliminary report. *Journal of Neuropsychiatry and Clinical Neurosciences*.

[41] Bosch MPC, van den Noort MWML, Bosch MPC, van den Noort MWML (2008). The search for the mechanism behind acupuncture: research with Neuroimaging. *Schizophrenia, Sleep, & Acupuncture*.

[42] World Health Organization (1992). *The ICD-10 Classification of Mental and Behavioural Disorders: Clinical Descriptions and Diagnostic Guidelines*.

[43] Beck AT, Steer RA, Brown GK (1996). *Manual For the Beck Depression Inventory-II*.

[44] Kay SR, Fiszbein A, Opler LA (1987). The positive and negative syndrome scale (PANSS) for schizophrenia. *Schizophrenia Bulletin*.

[45] World Medical Organization (2008). Declaration of Helsinki. http://www.wma.net/en/30publications/10policies/b3/index.html.

[46] Buysse DJ, Reynolds CF, Monk TH, Berman SR, Kupfer DJ (1989). The Pittsburgh Sleep Quality Index: a new instrument for psychiatric practice and research. *Psychiatry Research*.

[47] Hametner E, Frauscher B, Högl B (2012). L01 sleep in patients with Huntington’s disease: an interim analysis. *Journal of Neurology, Neurosurgery and Psychiatry with Practical Neurology*.

[48] Doi Y, Minowa M, Uchiyama M (2000). Psychometric assessment of subjective sleep quality using the Japanese version of the Pittsburgh Sleep Quality Index (PSQI-J) in psychiatric disordered and control subjects. *Psychiatry Research*.

[49] Backhaus J, Junghanns K, Broocks A, Riemann D, Hohagen F (2002). Test-retest reliability and validity of the Pittsburgh Sleep Quality Index in primary insomnia. *Journal of Psychosomatic Research*.

[50] Tsai PS, Wang SY, Wang MY (2005). Psychometric evaluation of the Chinese version of the Pittsburgh Sleep Quality Index (CPSQI) in primary insomnia and control subjects. *Quality of Life Research*.

[51] Yu H, Liu Y, Li S, Ma X (2009). Effects of music on anxiety and pain in children with cerebral palsy receiving acupuncture: a randomized controlled trial. *International Journal of Nursing Studies*.

[52] Ronan P, Robinson N, Harbinson D, MacInnes D (2011). A case study exploration of the value of acupuncture as an adjunct treatment for patients diagnosed with schizophrenia: results and future study design. *Journal of Chinese Integrative Medicine*.

[53] Cui C-L, Wu L-Z, Luo F (2008). Acupuncture for the treatment of drug addiction. *Neurochemical Research*.

[54] Schroer S, Adamson J (2011). Acupuncture for depression: a critique of the evidence base. *CNS Neuroscience and Therapeutics*.

[55] MacPherson H (2000). Out of the laboratory and into the clinic: acupuncture research in the real world. *Clinical Acupuncture and Oriental Medicine*.

